# Sequential Fuzzy Diagnosis Method for Motor Roller Bearing in Variable Operating Conditions Based on Vibration Analysis

**DOI:** 10.3390/s130608013

**Published:** 2013-06-21

**Authors:** Ke Li, Xueliang Ping, Huaqing Wang, Peng Chen, Yi Cao

**Affiliations:** 1 School of Mechanical Engineering, Jiangnan University, 1800 Li Hu Avenue, Wuxi 214122, Jiangsu, China; E-Mails: dayanlv@live.cn (K.L.); ping@jiangnan.edu.cn (X.P.); caoyi@jiangnan.edu.cn (Y.C.); 2 School of Mechanical and Electrical Engineering, Beijing University of Chemical Technology, 15 Beisanhuan East Road, Chao Yang District, Beijing 100029, China; E-Mail: wanghq_buct@hotmail.com; 3 Department of Environmental Science and Engineering, Mie University 1577 Kurimamachiya-cho, Tsu-shi, Mie-ken 514-8507, Japan; E-Mail: chen@bio.mie-u.ac.jp

**Keywords:** condition diagnosis, relative crossing information (RCI), pseudo Wigner-Ville distribution (PWVD), ant colony optimization (ACO), synthesizing symptom parameter (SSP)

## Abstract

A novel intelligent fault diagnosis method for motor roller bearings which operate under unsteady rotating speed and load is proposed in this paper. The pseudo Wigner-Ville distribution (PWVD) and the relative crossing information (RCI) methods are used for extracting the feature spectra from the non-stationary vibration signal measured for condition diagnosis. The RCI is used to automatically extract the feature spectrum from the time-frequency distribution of the vibration signal. The extracted feature spectrum is instantaneous, and not correlated with the rotation speed and load. By using the ant colony optimization (ACO) clustering algorithm, the synthesizing symptom parameters (SSP) for condition diagnosis are obtained. The experimental results shows that the diagnostic sensitivity of the SSP is higher than original symptom parameter (SP), and the SSP can sensitively reflect the characteristics of the feature spectrum for precise condition diagnosis. Finally, a fuzzy diagnosis method based on sequential inference and possibility theory is also proposed, by which the conditions of the machine can be identified sequentially as well.

## 1. Introduction

Motor systems are widely employed in modern industry. They convert produced electricity into other forms of energy to provide power to other equipment. Three-phase induction motors (IMs) are frequently adopted, mainly because of their low price, ruggedness, simplicity of control, and reliability. In practical applications, IM failures may cause the breakdown of equipment, and further, serious consequences may arise due to these failures. Thus, fault diagnosis and condition discrimination of IMs have an important significance for safe operation, guaranteeing production efficiency and reducing maintenance costs. Many reliability survey papers deal with failure statistics of electric machine subassemblies, focusing mainly on induction machines because of their widespread use in industry [1–5]. A rough classification identifies four classes of faults: bearing faults, stator-related faults, rotor-related faults, and other faults (cooling, connection, and terminal boxes). Among these, bearing faults account for over 40% of the total faults in electric machines, and other faults arising in electric machines are often associated with bearing faults. In many instances, the accuracy of the instruments and devices used to monitor and control the electric machines is highly dependent on the dynamic performance of bearings.

Vibration diagnosis is commonly used to detect the faults and identify the status of electric machines. Vibration diagnosis depends largely on the feature analysis of vibration signals measured for condition diagnosis, as these signals carry dynamic information about the machine status. Some methods based on the analysis of vibration signals have been investigated for bearing fault diagnosis [6–9]. In [6], the detection of damages of the bearings in servo motors was achieved by analyzing the frequency response. In [7], the authors developed a fault-signature model and a fault-detection scheme for using machine vibrations to detect inner-race defects. In [8], an amplitude modulation detector was designed to identify bearing faults in machine vibration data. In [9], the authors provided an overview of vibration signal processing techniques commonly used for bearing fault detection purposes. However, these techniques are all based on stationary analysis, but there are many industrial applications in which diagnostic approaches based on stationary analysis do not lead to satisfactory results. This is the case of applications in which the operation regime varies continuously or the diagnostic signal suffers perturbations. In such situations, other signal processing methods must be developed such as time–frequency analysis [10].

The Wigner-Ville distribution (WVD) is an important quadratic form time–frequency distribution with optimized resolution in both the time and frequency domains. The WVD has proven to be an effective tool for analyzing the behavior of non-stationary signals. It has been applied to fault diagnosis and condition monitoring in various machines [11–16]. In [11], the pseudo-WVD (PWVD) is applied to fault diagnosis in motor bearing. Reference [12] examines whether acoustic signals can be used to effectively detect the various local faults in gearboxes using the smoothed PWVD. Reference [13] applies a PWVD to identify the influence of the fluctuating load conditions on a gearbox. In [14], the WVD is applied to detect the presence of short-circuits in the electric machines. In [15], the WVD is applied to the diagnosis of mixed eccentricities in induction motors operating under various conditions. Rotor faults in brushless DC motors are detected during transients using WVD in [16].

When a computer is used for machine condition diagnosis, symptom parameters (SPs) are required to express the information indicated by a signal measured for diagnosing machinery faults [17]. However, under variable operating conditions, the signals measured from machines under different conditions often contain strong noises. Hence, the value of the SP calculated by these signals is ambiguous, and the diagnostic sensitivity of the SP is low. In this study, to increase the diagnostic sensitivity of the SP, we propose a method for obtaining the SSP by using the ACO clustering algorithm.

The ACO is a multiagent approach for solving combinatorial optimization problems. In ACO, artificial ant colonies cooperate in finding good solutions for difficult discrete optimization problems. ACO has been applied to a variety of different problems, such as scheduling [18], the traveling salesman problem [19], and timetabling [20]. Recently, ACOs have also entered the data mining domain, addressing both classification [21,22], and the clustering task [23,24], which is the topic of interest in this paper. In this study, a clustering model is constructed by using the ACO clustering algorithm. It is used to classify the SPs calculated from the signals in each machine state for condition diagnosis, as well as obtaining their optimal clustering centers. By using these clustering centers, a new coordinate axis is constructed. The SP is then projected to the new coordinate axis, and the SSP can be obtained. The diagnostic sensitivity of the SSP is greater than the original SP, and the conditions of the machine can be identified more easily.

In this paper, a novel condition diagnosis method for an electric motor which operates under unsteady conditions is proposed. The integration of the PWVD with the RCI methodology is used for extracting the feature spectra from the non- stationary vibration signal. The RCI allows us to automatically extract feature spectrum from the time-frequency distribution of the vibration signal, and the extracted feature spectrum is instantaneous and not correlated with the rotation speed and load. Moreover, the SSP, which has high sensitivity for condition diagnosis, is obtained by using the ACO clustering algorithm. The excellent SSP obtained by ACO can sensitively reflect the characteristics of the feature spectrum for precise diagnosis. In this paper, a fuzzy diagnosis method based on sequential inference and possibility theory is also proposed, by which the conditions of the machine can be identified sequentially.

This paper is organized as follows: in Section 2, the feature extraction method based on the PWVD and the RCI techniques is described. In Section 3, the six SPs for condition diagnosis are defined and a detection index (DI) using statistical theory has also been defined to evaluate the applicability of the SP. In Section 4, the ACO clustering model is constructed and the SSP, which has high sensitivity for condition diagnosis, is obtained. In Section 5, a fuzzy diagnosis method based on sequential inference and possibility theory is proposed. In Section 6, the experimental setup is described and a practical example of condition diagnosis for a motor bearing is presented. Section 7 summarizes and concludes this paper.

## 2. Extraction of Features

### 2.1. Wigner-Ville Distribution (WVD)

The WVD is an important quadratic-form time–frequency distribution which provides high resolution. WVD possesses a great number of good properties, and it has wide interest for fault detection with non-stationary signal analysis [25]. However, an important drawback of the distribution is its nonlinearity due to the quadratic nature. If the analyzed signal contains more than one frequency component, the WVD method suffers from cross-term interference, resulting in difficulties for discriminating the actual frequency components.

There are several proposed techniques that try to suppress the cross-terms at the expense of the loss of time-frequency resolution. A usual way is to use a windowed version of WVD obtaining the so-called pseudo Wigner-Ville distribution (PWVD):

(1) $$\text{PWV}D_{x}(t,\omega )=\frac{1}{2\pi }\int_{-\infty }^{+\infty }x^{*}\left(t-\frac{1}{2}\tau \right)x\left(t+\frac{1}{2}\tau \right)h\left(\tau \right)e^{-j\tau \omega }d\tau$$

where *ω* is an angular frequency, the symbol * denotes the complex conjugate, *h*(*τ*) is the window function, and the Gaussian window is determined in this study. The real PWVD examples are shown in Section 6 (refer to Figures 11, 12, 13 and 14(b)).

### 2.2. Feature Spectrum Extracted by RCI

After transforming the signal into the time-frequency domain using the PWVD, the instantaneous feature spectra need to be automatically extracted by computer for intelligent fault diagnosis. In this paper, the RCI is proposed to automatically extract the feature spectra from the PWVD to identify the machine's condition. In our early works the RCI has been successfully applied to extract the feature spectra from various time-frequency analyses such as instantaneous power spectrum (IPS) [26] or short time Fourier transform (STFT) [27].

**Figure 1.**
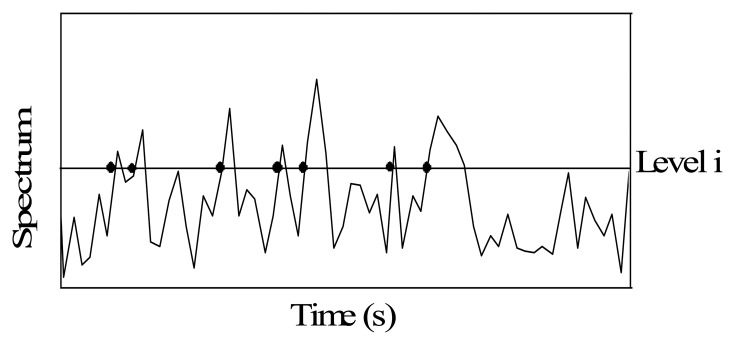
Frequency of crossing over level *i*.

As shown in Figure 1, *q_i_*(*t*) is the number of crossings over of some level *i* of the vertical coordinate of the spectrum *P*(*t*,*ω*) with a positive slope in unit time and can be calculated as follows [28]:

(2) $$q_{i}(t)=\frac{\sigma_{v}(t)}{2\pi \sigma_{x}(t)}\exp\left(-{\left\{i/2\sigma_{x}(t)\right\}}^{2}\right)$$

(3) $$\sigma_{x}{\left(t\right)}^{2}=\int_{0}^{\infty }P(t,\omega )d\omega$$

(4) $$\sigma_{v}{\left(t\right)}^{2}=\int_{0}^{\infty }{\left(\omega \right)}^{2}P(t,\omega )d\omega .$$

The vertical coordinate axis of a spectrum in the normal state is divided into *M* equal sections from the spectrum's maximum amplitude to its minimum amplitude. *q_ni_*(*t*) and *q_ai_*(*t*) can be calculated by Equation (2) in section *i* (*i* = 1∼*M*) in the normal state and the state to be detected, respectively. Here, *n* and *a* indicate normal state and the state to be detected, respectively. The RCI is expressed by *I_q_*(*t*) and is defined as follows:

(5) $$I_{q}(t)=\overset{M}{\underset{i=1}{\text{∑}}}\left|\log\left(q_{at}(t)/\bar{q_{ni}}\right)\left(q_{at}(t)-\bar{q_{ni}}\right)\right|$$

where $\bar{q_{ni}}=\frac{1}{T}\int_{0}^{T}q_{ni}(t)dt$ and *T* is the sampling time.

If the spectrum is measured in an abnormal state, *q_ni_* and *q_ai_* differ and the value of *I_q_* increases. Thus, *I_q_* can be used to express the difference in spectra between the normal and abnormal states, by which the feature spectra can be extracted in the time-frequency domain.

## 3. SP and Sensitivity Evaluation

### 3.1. SP for Fault Diagnosis

After the feature spectra are extracted from the PWVD by the RCI, in order to automatically diagnose the machine states by computer, the SPs calculated from the RCI are defined to express the characteristics of the feature spectra.

A good SP can correctly reflect the states and condition trends of machines. Many SPs have been defined in the pattern recognition field. This study considers the following six non-dimensional SPs in the frequency domain that are commonly used to diagnose machine faults.

Frequency-domain Skewness:

(6) $$P_{1}=\frac{\overset{I}{\underset{i=1}{\text{∑}}}{\left(f_{i}-\bar{f}\right)}^{3}\cdot F(f_{i})}{\sigma^{3}I}$$

Frequency-domain kurtosis:

(7) $$P_{2}=\frac{\overset{I}{\underset{i=1}{\text{∑}}}{\left(f_{i}-\bar{f}\right)}^{4}.F(f_{i})}{\sigma^{4}\cdot I}.$$

Mean frequency that wave shape cross the mean of time-domain signal:

(8) $$P_{3}=\sqrt{\frac{\overset{I}{\underset{i=1}{\text{∑}}}f_{i}^{4}\cdot F(f_{i})}{\overset{I}{\underset{i=1}{\text{∑}}}f_{i}^{2}\cdot F(f_{i})}.}$$

Stabilization factor of wave shape:

(9) $$P_{4}=\frac{\overset{I}{\underset{i=1}{\text{∑}}}f_{i}^{2}\cdot F(f_{i})}{\sqrt{\overset{I}{\underset{i=1}{\text{∑}}}F(f_{t})\overset{I}{\underset{i=1}{\text{∑}}}f_{i}^{4}\cdot F(f_{i})}}.$$

Sum of the squares of the power spectrum:

(10) $$P_{5}=\overset{I}{\underset{i=1}{\text{∑}}}F^{2}(f_{i}).$$

Square root of the sum of the squares of the power spectrum:

(11) $$P_{6}=\sqrt{\overset{I}{\underset{i=1}{\text{∑}}}F^{2}(f_{i})}$$

where I is the number of spectra, *f_i_* is the value of the analysis frequency, *F*(*f_i_*) is the spectrum value of *f_i_*, *f̅* is the mean value of the analysis frequency, where $\bar{f}=\frac{\overset{I}{\underset{i=1}{\text{∑}}}f_{i}\cdot F(f_{i})}{\overset{I}{\underset{i=1}{\text{∑}}}F(f_{i})},$ *σ* is the standard deviation and $\sigma =\sqrt{\frac{\overset{I}{\underset{i=1}{\text{∑}}}{\left(f_{i}-\bar{f}\right)}^{2}\cdot F(f_{i})}{I}}$.

### 3.2. DI for Evaluating Symptom Parameters

For automatic diagnosis, SPs that can sensitively distinguish the fault types are needed. In order to evaluate the sensitivity of an SP for distinguishing two states, such as a normal or an abnormal state, DI is defined as follows. Supposing that *x*_1_and *x*_2_ are the SP values calculated from the signals measured in state 1 and state 2 respectively, and their average and standard deviation are μ and σ. The DI is calculated by:

(12) $$DI=\frac{\left|\mu_{1}-\mu_{2}\right|}{\sqrt{\sigma_{1}+\sigma_{2}}}.$$

The Distinction Rate (DR) is defined as:

(13) $$DR=1-\frac{1}{\sqrt{2\pi }}\int_{-\infty }^{-DI}\exp(-\frac{\mu^{2}}{2})d_{\mu }.$$

It is obvious that the larger the value of the DI, the larger the value of the DR will be, and therefore, the better the SP will be. Thus, the DI can be used as the index of the quality to evaluate the distinguishing sensitivity of the SP.

## 4. Obtain SSP by ACO Clustering Algorithm

In this paper, the SPs in the frequency domain are used to reflect the characteristics of the feature spectra extracted from the time-frequency distribution of the vibration signals. However, in most condition diagnosis cases, the values of SPs calculated from the vibration signals for condition monitoring and fault diagnosis are ambiguous, and the diagnostic sensitivity of the SPs is low. The main reasons for this can be explained as follows: (1) when the rotation speed and load of a machine vary while vibration signals is being measured and a fault is in an early stage, the signal contains strong noise, stronger than the actual failure signal, which may lead to misrecognition of useful diagnostic information. (2) The statistical objectivity of the measured signal cannot always be satisfied because of the measuring techniques and manner of the inspectors.

In this paper, we propose a new method for automatically obtaining the SSP by the ACO clustering algorithm. The ACO clustering algorithm can easily classify the SPs calculated from the signals in each machine state for condition diagnosis as well as obtain their optimal clustering centers. By using these clustering centers, a new coordinate axes is constructed. The SP is then projected to the new coordinate axes, by which the SSP can be obtained. This method aims to improve the sensitivity of the SP and increase class separability. The sensitivity of the SSP is greater than the original SP, and the conditions of the machine can be identified more easily.

### 4.1. ACO Clustering Algorithm

Assume that *N* is the sample set of vibration signals measured in *m* different states, the length of *N* is *n*, and *N* = {*x_1_, x_2_*…*x_n_*}. Every sample signal has *t* identified symptoms (in this paper, the symptoms are P_1_−P_6_). Then, the clustering analysis is to divide *n* sample data into *m* states, such that the objective function *F* shown in Equation (15) is minimized:

(15) $$\min F=\overset{m}{\underset{j=1}{\text{∑}}}\overset{n}{\underset{i=1}{\text{∑}}}{\overset{t}{\underset{k=1}{\text{∑}}}a_{ij}\left\|x_{ik}-c_{jk}\right\|}^{2}$$

where *c_jk_* is the clustering center, and:

(16) $$c_{jk}=\frac{\overset{n}{\underset{i=1}{\text{∑}}}a_{ij}x_{ik}}{\overset{n}{\underset{i=1}{\text{∑}}}a_{ij}}\;(j=1,2\cdots m;k=1,2\cdots t)$$

(17) $$a_{ij}=\{\begin{array}{c} 1\;if\;x_{i}\in \text{statej}\\ 0\;if\;x_{i}\in \text{statej} \end{array}(i=1,2\cdots n;j=1,2\cdots m).$$

In this paper, the procedure for applying the ACO for the condition diagnosis is proposed, and the procedure is explained as follows:

1) The SPs used for reflecting the features of the sample signals are input into the ACO algorithm.
2) The sample signals are randomly classified by artificial ants (artificial ants construct solutions), and the pheromone matrix that represents information between sample data and the clustering centers is initialized.
3) According to the solutions, the clustering centers are calculated by Equation (16), and the object function of each solution is calculated by Equation (15).
4) A local search is performed.
5) The pheromone matrix is updated.
6) According to the pheromone matrix, artificial ants update the solutions.
7) Steps (3)−(6) are looped until the ending condition is satisfied.

In the ACO, each artificial ant will construct the solution *S* with a length of *n; S* = {*c_i_*|*i* = 1, 2 … *n*}, and *c_i_ =* 1,2…*m*, where *c_i_* is the classification result of sample *x_i_*. That is, if *c_i_ = j*, then *x_i_* is the output vibration data in state *j*. At the start of the ACO, the solutions *S* are randomly constructed by artificial ants, and with the increase of the iteration number, artificial ants continuously update the solutions according to the pheromone matrix information, followed by the principles given as follows:

(18) $$S=\arg\max\left\{\tau_{ij}\times {\left[\frac{1}{d_{ij}}\right]}^{\beta }\right\}\text{if}\;q\leq q_{o}$$

where *d_ij_* is the Euclidean distance between clustering center *j* and sample *x_i_* $d_{ij}=\sqrt{\overset{t}{\underset{k=1}{\text{∑}}}{\left(x_{ik}-c_{jk}\right)}^{2},}$ where *q* is a value chosen randomly with a uniform probability between 0 and 1, *q_o_* is constant, 0 *< q_o_<* 1,***τ****_ij_* represents the pheromone concentration of sample *x_i_* associated with the state *j* and β is a parameter that determines the relative importance of heuristic information (the choice of β is determined experimentally, where β *>* 0).

If *q_o_< q*, the artificial ants select the state for sample *x_i_* by the conversion probability *p_ij_* given as follows:

(19) $$p_{ij}=\frac{\tau_{ij}\times \left[\frac{1}{d_{ij}}\right]}{{\overset{m}{\underset{s=1}{\text{∑}}}\tau_{is}\times \left[\frac{1}{d_{ij}}\right]}^{\beta }}.$$

To improve the efficiency and accelerate the convergence speed of the ACO, the method of local search for the ACO is presented. The local search method is conducted on all solutions or some solutions [29]. In this paper, the latter is applied, that is, local search is only implemented for the ten solutions with smaller objective functions. The execution process of the local search for the ACO is as follows:

1) All of the solutions are arranged in ascending order according to the values of the objective function.
2) Random data *W_i_*{ *i =* 1,2…*n* } for every sample are produced automatically.
3) A weight *P* is set, where 0 < *P <* 1.
4) *P* is compared with *W_i_*, if *P > W_i_*, and then the sample *x_i_* is reclassified.
5) The Euclidean distance between sample *x_i_* and each clustering center is calculated, and sample *x_i_* is reclassified into the class with the shortest Euclidean distance.
6) Equation (15) is used to compute the objective function again and compare it with the former objective function values. If the new one is lower than the former one, the new solution sets are kept; if the new one is greater than the former one, the former solution sets are kept.
7) Steps (2)–(6) are looped until the ten solutions are calculated.

After performing the local search operation, the pheromone matrix is updated. Such a pheromone updating process reflects the usefulness of dynamic information provided by the artificial ants. Formally, pheromone trails are updated by following rule:

(20) $$\tau_{ij}(l+1)=(1-\rho )\tau_{ij}(l)+\overset{10}{\underset{a=1}{\text{∑}}}\Delta \tau_{ij(a)}$$

(21) $$\Delta \tau_{ij(a)}=\{\begin{array}{ll} \frac{1}{F_{a}} & x_{i}\in \text{statej}\\ 0 & \text{otherwise} \end{array}$$

(22) $$F_{a}=\overset{m}{\underset{j=1}{\text{∑}}}\overset{n}{\underset{i=1}{\text{∑}}}{\overset{t}{\underset{k=1}{\text{∑}}}\left\|x_{ik}-c_{jk}\right\|}^{2}$$

where *τ_ij_* represents the pheromone concentration of sample *x_i_* associated with state *j,ρ* is the decay parameter of the pheromone and, to prevent pheromone excessive accumulation 0 < *ρ* < 1, Δ*τ_ij_*_(_*_a_*_)_ is the pheromone values of artificial ant *a*.

In order to explain the process of the optimum clustering centers are obtained by the ACO clustering algorithm, an example that obtains the optimum clustering centers of a bearing in normal and roller defect states is given as follows.

**Figure 2.**
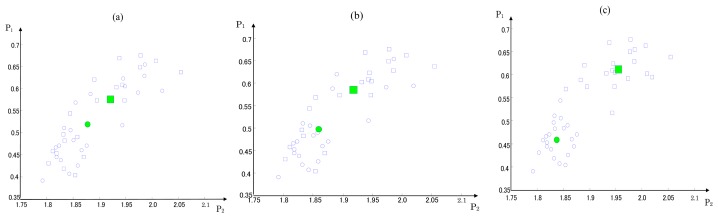
Changes in the clustering centers: (**a**) at the start of the ACO, (**b**) after 100 iterations and (**c**) after 200 iterations. The symbols ○ and □ express the value samples of the SPs in the normal and roller defect states, respectively, and the large symbols ■ and ● represent their respective clustering centers.

As shown in Figure 2(a)–(c), the sample data are first randomly classified into the normal and roller defect states. The clustering centers and the sum of the spatial distance between all of the sample data and the clustering centers are calculated by Equations (15)–(17). As the iteration number increases, the pheromone is continuously updated, and the classification of the sample data and clustering centers are also updated by the artificial ants according to the pheromone information. After approximately 200 iterations, the ACO converged to the optimum clustering centers, and the optimal clustering centers are calculated with the minimum sum of the spatial distances.

### 4.2. SSP

After obtaining the clustering centers, the SSPs can be obtained by these clustering centers and a projection method. The execution process is as follows.

**Figure 3.**
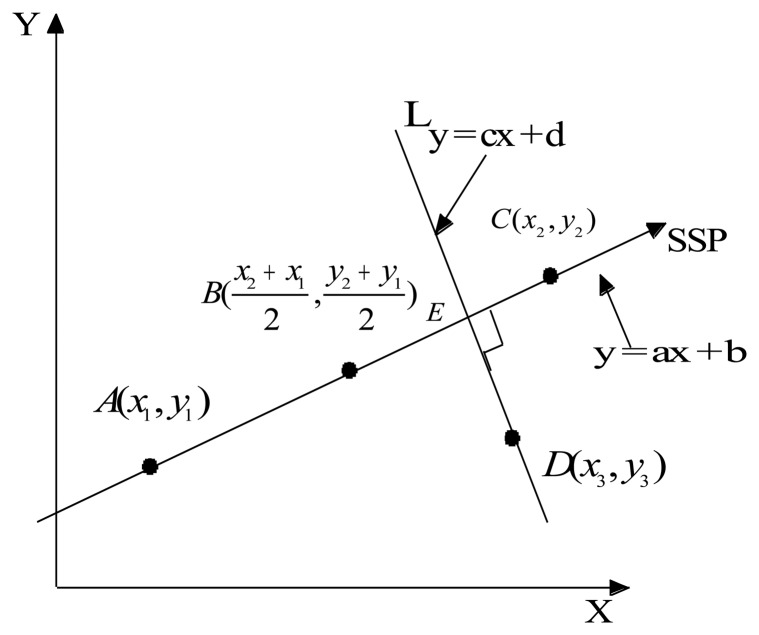
The axis of the synthetic symptom parameter (SSP).

Assuming that *x* and *y* are values of the two SPs calculated from the signals measured in states 1 and 2, respectively, as shown in Figure 3, then A and C are the clustering centers of *x* and *y* in states 1 and 2, respectively. The SSP axis is defined as passing through points A and C. The coordinate values of A and C are (*x*_1_, *y*_1_) and (*x*_2_,*y*_2_), respectively. B is the center point between A and C, and the coordinate values of B are $\frac{x_{2}+x_{1}}{2},\frac{y_{2}+y_{1}}{2}.$ The line *L* is passing through the arbitrary point D (*x*_3_,*y*_3_) and perpendicular to the SSP line. The coordinate value of the intersecting point E between the SSP axis and line *L* is obtained as follows:

Linear equation of the SSP axis:

(23) y=ax+b

Linear equation of line *L:*

(24) y=cx+d

where $c=-\frac{1}{a}\text{and}d=y_{3}+\frac{1}{a}x_{3}$.

Point E is calculated using Equations (23) and (24) and is expressed as follows:

$$(\frac{ay_{3}+x_{3}-ab}{a^{2}+1},\frac{a^{2}y_{3}+ax_{3}+b}{a^{2}+1}).$$

We define point B as the origin coordinate of the SSP axis, and the distance between B and E is:

(25) $$D_{BE}=\sqrt{{\left(\frac{ay_{3}+x_{3}-ab}{a^{2}+1}-\frac{x_{2}+x_{1}}{2}\right)}^{2}+{\left(\frac{a^{2}y_{3}+ax_{3}-b}{a^{2}+1}-\frac{y_{2}+y_{1}}{2}\right)}^{2}.}$$

According to the previous discussions, we can define a new synthetic symptom parameter (SSP) as follows:

(26) $$\text{SSP}=m\sqrt{{\left(\frac{aP_{j}+P_{i}-ab}{a^{2}+1}-\frac{x_{2}+x_{1}}{2}\right)}^{2}+{\left(\frac{a^{2}P_{1}+aP_{1}-b}{a^{2}+1}-\frac{y_{2}+y_{1}}{2}\right)}^{2}}$$

where *P_i_* and *P_j_* are the SPs calculated from the vibration signals measured in each state; $a=\frac{y_{2}-y_{1}}{x_{2}-x_{1}}$ and $b=\frac{x_{2}y_{1}-x_{1}y_{2}}{x_{2}-x_{1}};$; *m* is a parameter that equals 1 if $\frac{aP_{j}+P_{i}-ab}{a^{2}+1}-\frac{x_{2}+x_{1}}{2}\geq 0$ and −1 if $\frac{aP_{j}+P_{i}-ab}{a^{2}+1}-\frac{x_{2}+x_{1}}{2}<0$.

**Figure 4.**
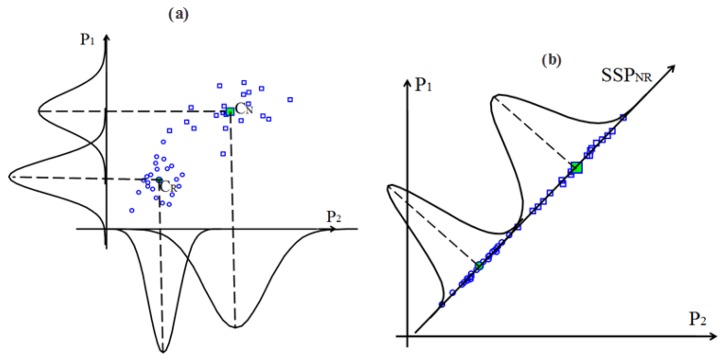
Illustrative example of an SSP: (**a**) probability distributions of P_1_and P_2_ and (**b**) probability distributions of SSP_NR_.

To explain the efficiency of the SSP, we provide some examples below. In the example shown in Figure 4, we used two SPs, P_1_ and P_2_ to distinguish the normal and roller element defect states. C_N_ and C_R_ are the clustering centers obtained by the ACO for the normal and roller element defect states, respectively. The SSP_NR_ can be obtained using C_N_, C_R_ and Equation (26). Figure 4(a) shows the probability distributions of P_1_and P_2_ in the normal and roller defect states, respectively. Figure 4(b) shows the probability distributions of SSP_NR_ in the normal and roller defect states, respectively.

From Figure 4, it is obvious that the overlap of the probability distributions of the SSP_NR_ is smaller than P_1_ and P_2_. A smaller overlap area indicates a higher sensitivity for distinguishing the two states and demonstrates that the symptom parameter is better. Thus, the sensitivity of the SSP_NR_ is higher than those of P_1_ and P_2_ for distinguishing the normal and roller element defect states, respectively.

## 5. Sequential Diagnosis Method Based on Possibility Theory

### 5.1. Possibility Theory

In the motor fault diagnosis, knowledge of fault diagnosis is incomplete and vague due to the complexity of the motor. One reason that causes the incompleteness is that we often do not have a complete set of parameters necessary to fully describe a faulty behavior or component. This uncertainty nature of the problem leads us to seek a solution in fuzzy diagnostic models.

Possibility theory is a mathematical theory for dealing with certain types of uncertainty and is an alternative to probability theory. The basic idea of possibility theory, introduced by Zadeh, is to use fuzzy sets not only to represent the gradual aspect of vague concepts such as “large”, but also to represent incomplete knowledge, tainted with imprecision and uncertainty [30,31]. Recently, possibility theory has been used for fault diagnosis [32,33]. In [32] and [33], possibility theory was applied to condition diagnosis in rotating machinery to process the uncertain relationship between the symptoms and fault types.

For fuzzy inference, membership functions of SP are necessary. These functions can be obtained from the probability density functions of the SP using possibility theory. When the probability density function of the SP conforms to the normal distribution, it can be changed to a possibility function *μ*(*x_i_*) using the following equation [32]:

(27) $$\mu (x_{i})=\overset{N}{\underset{k=1}{\text{∑}}}\min\left\{\lambda_{i},\lambda_{k}\right\}$$

λ*_i_* and λ*_k_* can be calculated as follows:

(28) $$\begin{array}{l} \lambda_{i}=\int_{x_{i-1}}^{x_{i}}\frac{1}{\sigma \sqrt{2\pi }}\exp\left\{-\frac{{\left(x-\bar{x}\right)}^{2}}{2\sigma^{2}}\right\}dx\\ \lambda_{k}=\int_{k_{i-1}}^{k_{i}}\frac{1}{\sigma \sqrt{2\pi }}\exp\left\{-\frac{{\left(x-\bar{x}\right)}^{2}}{2\sigma^{2}}\right\}dx \end{array}$$

where *x* is the value of SP, and $x\in \left[\bar{x}-3\sigma ,\bar{x}+3\sigma \right]$; σ and *x̅* are the standard deviation and mean of *x*, respectively.

**Figure 5.**
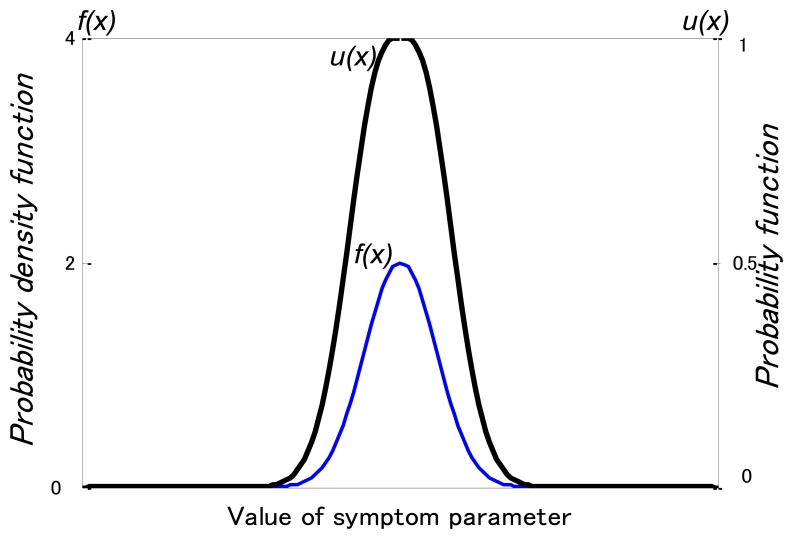
Possibility and probability density functions.

**Figure 6.**
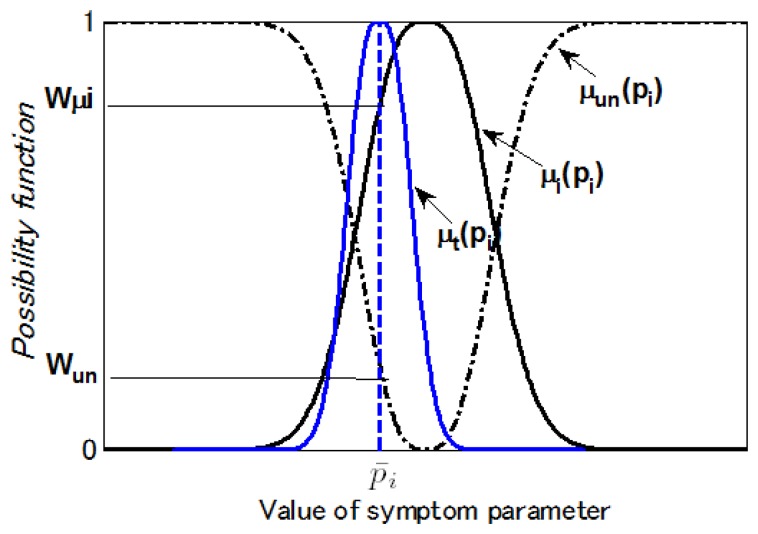
Matching examples of the possibility function.

Figure 5 illustrates the possibility and probability density functions. Figure 6 shows the matching examples of the possibility function. *p_i_* is a SP for diagnosing state *i*. The possibility function *μ_i_*(*p_i_*) in the state *i* can be easily calculated as Figure 6. The possibility function of the SP in other states expressed with *μ_un_* (*p_i_*), and calculated by:

(29) $$\mu_{i}(p_{i})+\mu_{un}(p_{i})=1.$$

If *μ_t_*(*p_i_*) is the possibility function calculated from the data in the state to be diagnosed, the match degrees with relevant level are calculated as follows:

(30) $$\begin{array}{l} \text{State}\;i\;\text{level}:W_{ui}=\mu_{ui}(p_{i})\wedge {\bar{p}}_{i}\\ \text{Other state level}:W_{un}=\mu_{un}(p_{i})\wedge {\bar{p}}_{i}. \end{array}$$

### 5.2. Sequential Condition Diagnosis Approach

**Figure 7.**
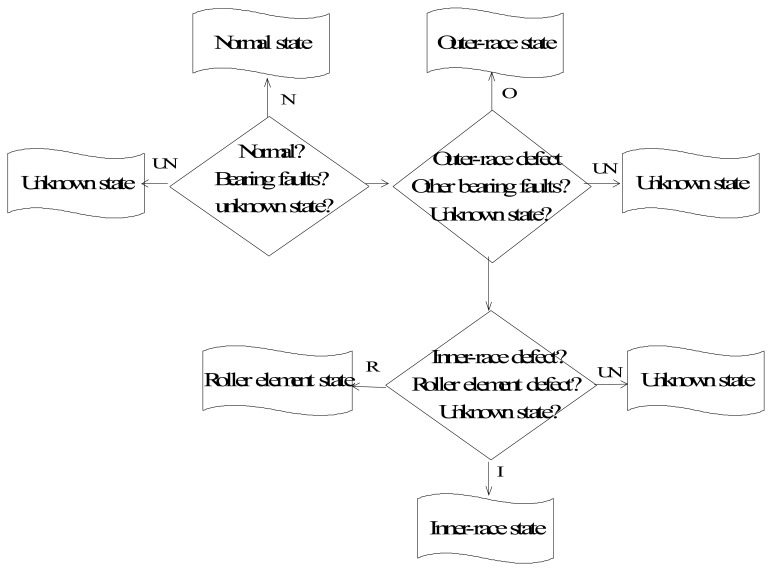
Flowchart of the sequential condition diagnosis.

In many cases of condition diagnosis, the symptom parameters are defined to reflect the features of vibration signals measured in each state in order to diagnose faults. However, it is difficult to find one symptom parameter or a few symptom parameters that can identify all of the faults simultaneously. In contrast, the SP for identifying two states is easily identified. To solve these problems, a sequential diagnosis method is proposed, as shown in Figure 7. The inference of sequential diagnosis is follows:

In the first step, the possibility grades of normal, bearing fault and unknown abnormal states are g_N_, g_A_ and g_UN_ respectively, if g_N_ > g_A_ and g_N_ > g_UN_ then the state is judged normal state, if g_UN_ > g_A_ and g_UN_ > g_N_, then the state is judged unknown abnormal state, else proceed to next step.

In the second step, the possibility grades of outer-race defect and other bearing fault states are g_o_ and g_B_ respectively, if g_O_ > g_B_ and g_O_ > g_UN_, then the state is judged outer-race defect, if g_UN_ > g_O_ and g_UN_ > g_B_, then the state is judged unknown abnormal state, else proceed to next step.

In the third step, the possibility grades of inner-race defect and roller element defect are g_I_ and g_R_ respectively, if g_I_ > g_R_ and g_I_ > g_UN_, then the state is judged inner-race defect state, if g_R_ > g_I_ and g_I_ > g_UN_, then the state is judged roller element defect state, else it will be judged as unknown abnormal state.

## 6. Diagnosis and Application

**Figure 8.**
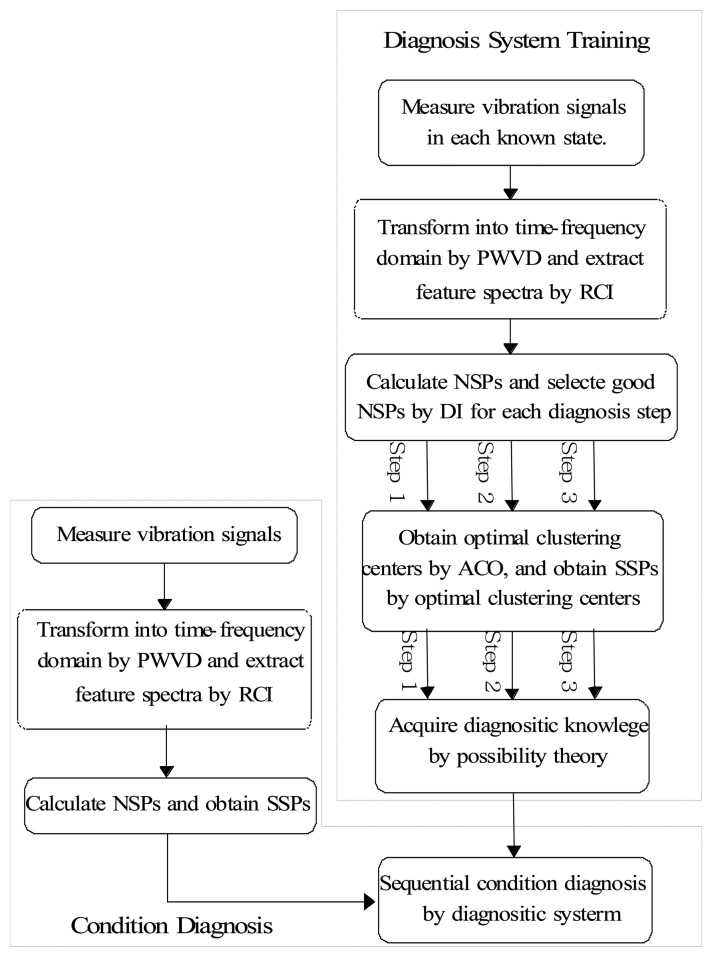
Flowchart for condition diagnosis.

In order to prove the feasibility of the proposed condition diagnosis algorithm, a practical diagnosis experiment is performed. In this section, the application of fault diagnosis to a motor bearing is presented. The flowchart of the fault diagnosis is shown in Figure 8. Firstly, the vibration signals in each beforehand known state is transformed by the PWVD technique, and the feature spectra are automatically extracted by the RCI. Secondly, the original SPs in the frequency domain are calculated by the extracted feature spectra, and the good SPs are selected by DI for each diagnosis step. Thirdly, the SSPs for each diagnosis step are obtained by the ACO clustering algorithm. Fourthly, using these SSPs, diagnostic knowledge (possibility distribution of each beforehand known state) is acquired by possibility theory. Lastly, conditions of a machine are automatically identified by diagnostic knowledge, when input the possibility distribution of the unknown state to be diagnosed.

### 6.1. Experimental System for Fault Diagnosis

**Figure 9.**
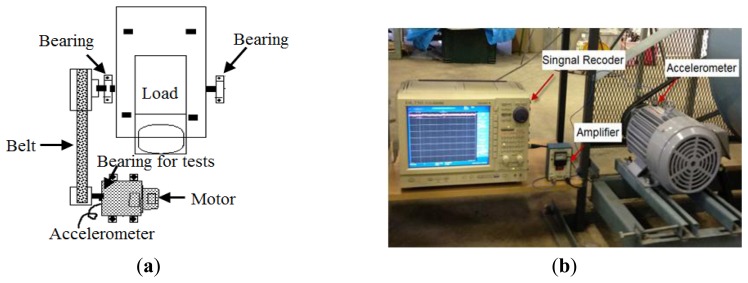
Experimental setup for the rolling bearing fault diagnosis. (**a**) Illustration of the rotation machinery and (**b**) the motor in the field.

In this paper, an inductor motor (Mitsubishi SB-JR) used in a centrifugal fan system is employed for the faults diagnosis test. The nameplate of the machine is 3.7 kW three-phase induction motor, with *V_max_* = 220V, *P* = 4 pole pairs, rated speed *S* = 1,800 rpm. Rated slip and frequency are 6.5% and 60 Hz. The rotor is carried by two bearings, one of which is defective. Figure 9 shows the Illustration and photo of the experimental setup for rolling bearing fault diagnosis.

The rotation speed varies from 400 to 800 rpm while the signals are being measured by changing the optional input voltage of the motor. The load, which is the torque exerted on the rotating shaft, is also varied by manually and randomly rubbing the timing belt between the motor and rotating shaft using an implement while the data was being measured.

In this study, an accelerometer (PCB MA352A60) with a bandwidth from 5 Hz to 60 kHz and a 10 mV/g output is used to measure the vertical vibration signals in the normal, outer-race defect, inner-race defect, and roller element defect states, respectively. The vibration signals measured by the accelerometer are transformed into the signal recorder (Scope Coder DL750) after being magnified by the sensor signal conditioner (PCB ICP Model 480C02). The sampling frequency of the signal measurement is 50 kHz, and the sampling time is 20 s.

**Figure 10.**
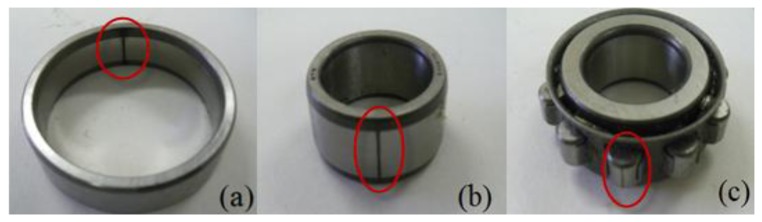
Bearing defects: (**a**) outer-race defect, (**b**) inner-race defect, and (**c**) roller element defect.

The most common faults in a roller element bearing are the outer-race defect, inner-race defect and roller element defect. In this study, to obtain the signals in the normal and the faults states of the bearing, two types of roller bearings (N205 and NU205) are used for the fault diagnosis test. The N205 with separable out-race is used for normal, outer-race defect and roller element defect states. The NU205 with separable inner-race is used for inner-race defect state. These fault bearings, which were artificially made using a wire-cutting machine, are shown in Figure 10. The specifications of the test bearings, size of the faults, and other necessary information are listed in Table 1. The signals measured for diagnosis are normalized using the following equation prior to application of the PWVD:

(31) $$x=\frac{x^{\prime }-\mu }{\sigma }.$$

### 6.2. Feature Extraction

**Figure 11.**
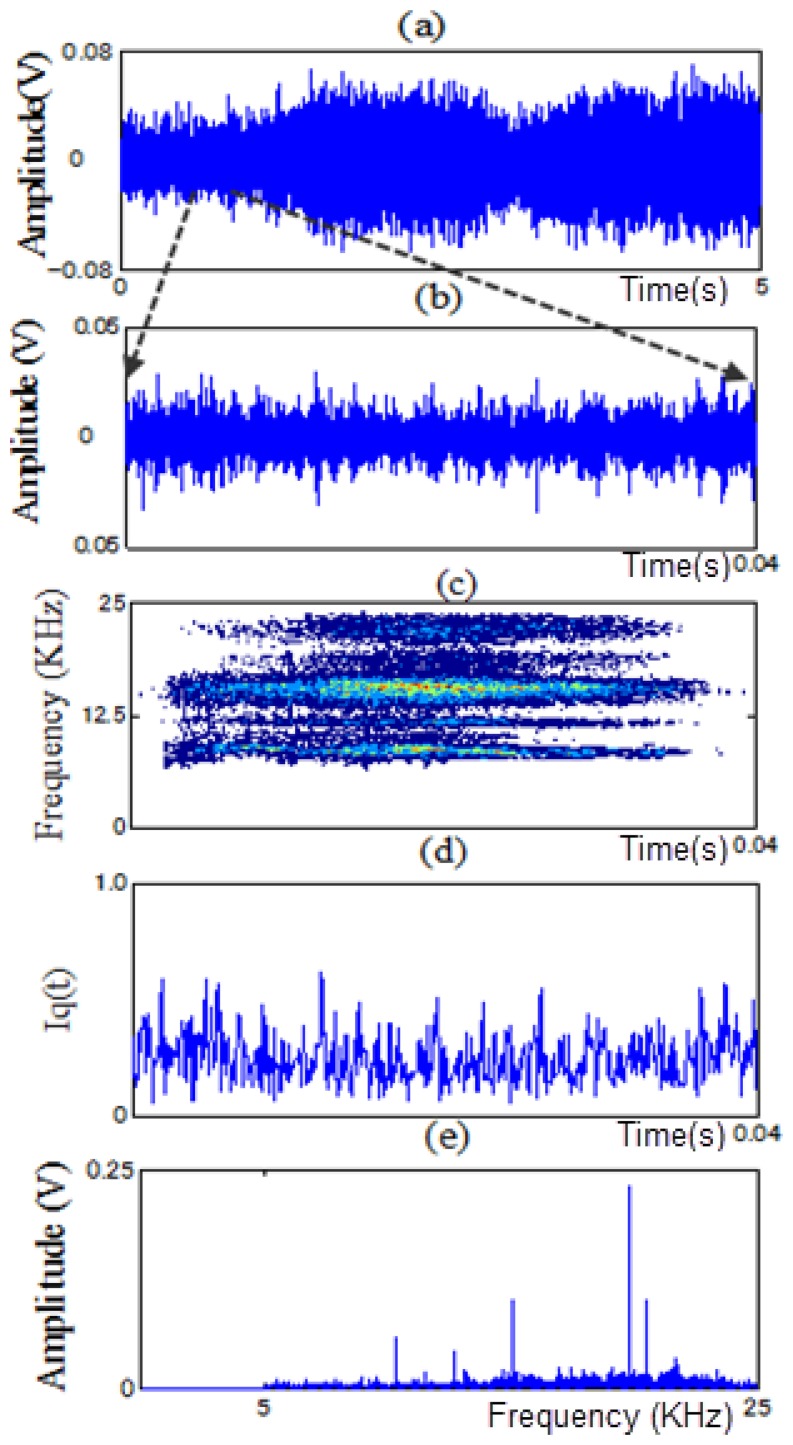
Contour graph and RCI of the spectra in the normal state: (**a**) time signal. (**b**) a part of time signal. (**c**) contour graph. (**d**) RCI and (**e**) power spectrum.

**Figure 12.**
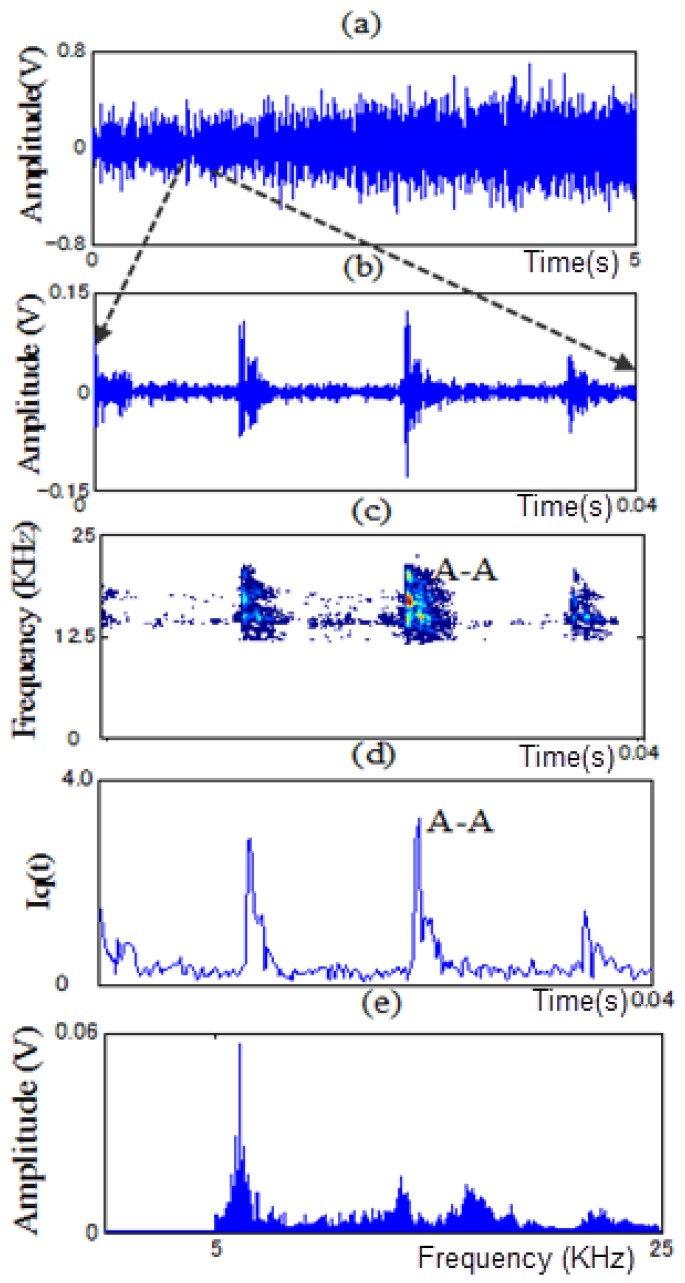
Contour graph and RCI of the spectra in the outer-race defect state: (**a**) time signal. (**b**) a part of time signal. (**c**) contour graph. (**d**) RCI and (**e**) power spectrum at position A-A.

**Figure 13.**
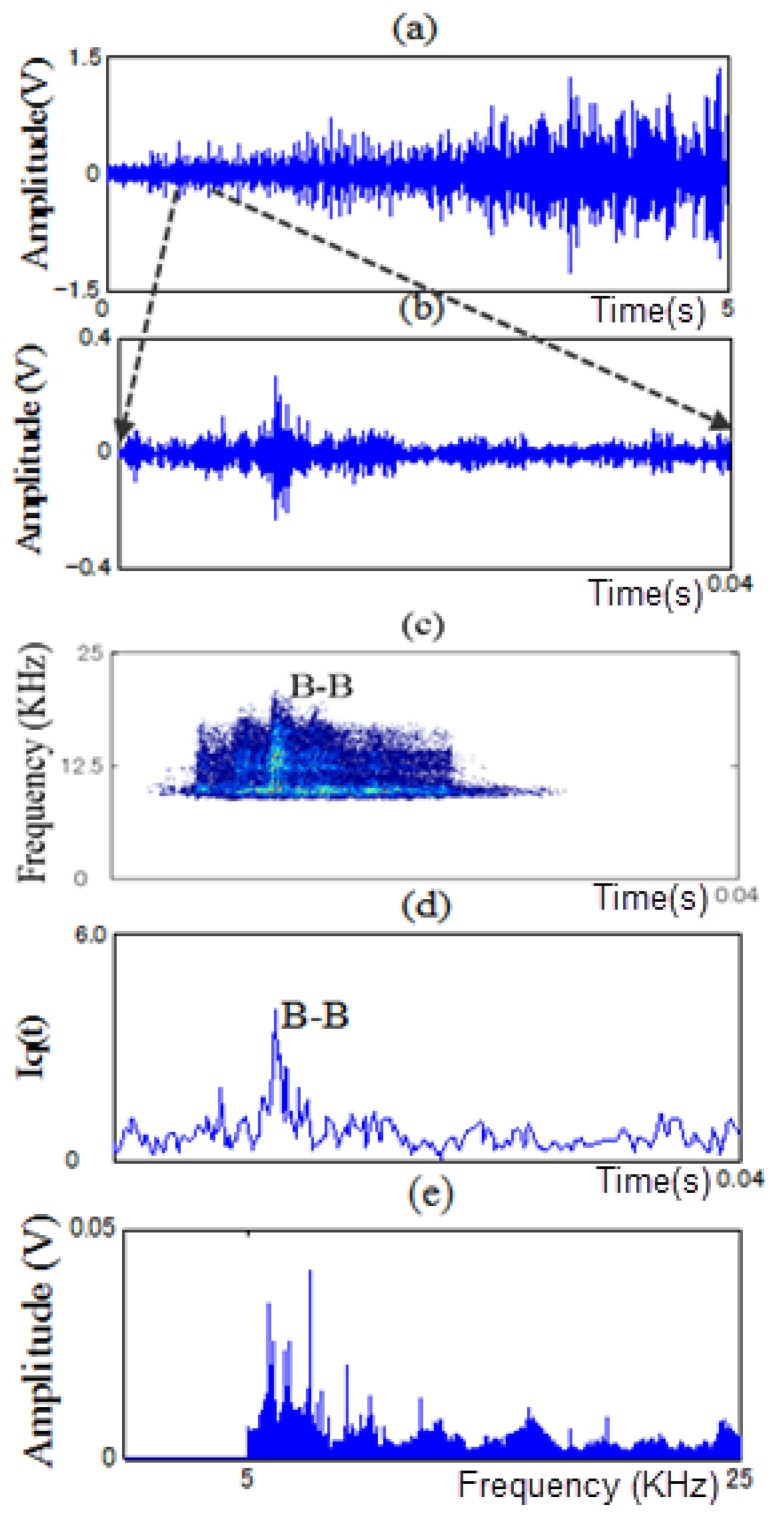
Contour graph and RCI of the spectra in the inner-race defect state: (**a**) time signal. (**b**) a part of time signal. (**c**) contour graph. (**d**) RCI and (**e**) power spectrum at position B-B.

**Figure 14.**
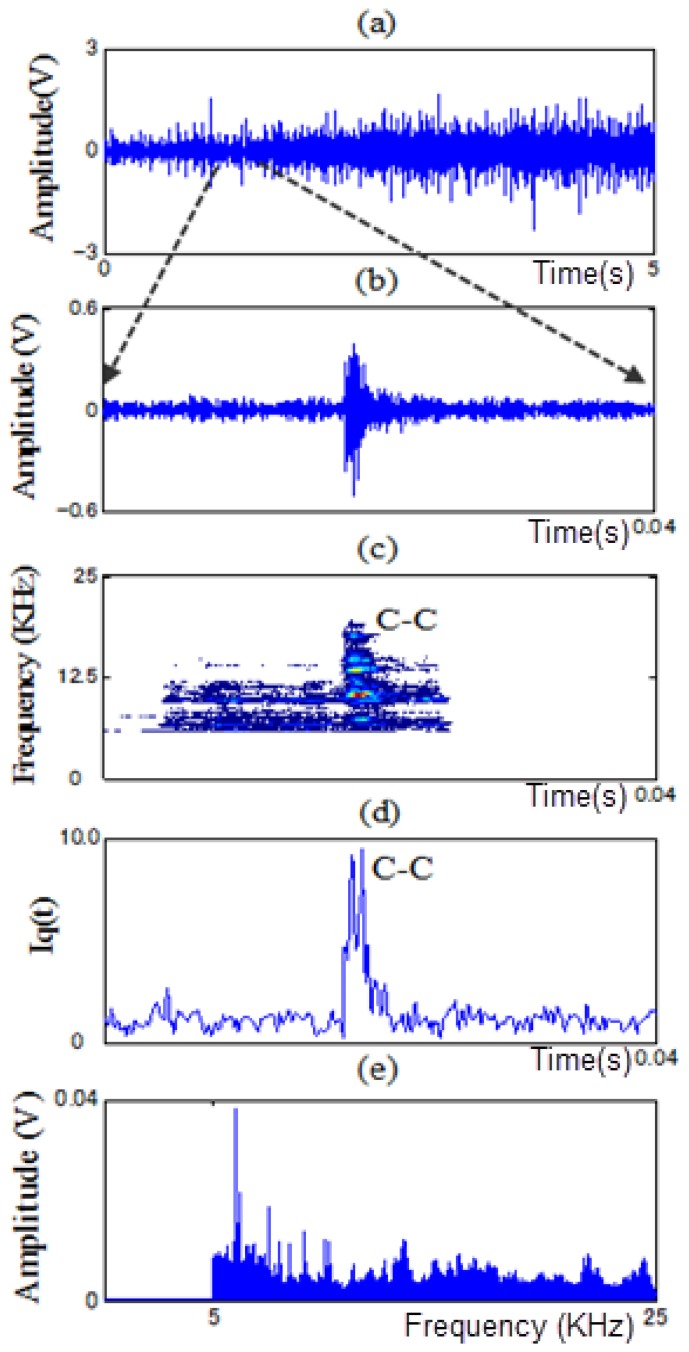
Contour graph and RCI of the spectra in the roller element defect state: (**a**) time signal, (**b**) a part of time signal, (**c**) contour graph, (**d**) RCI, and (**e**) power spectrum at the position C-C.

In this paper, PWVD and RCI methods are proposed to extract features from non-stationary vibration signals to identify the fault types of a motor bearing under variable operating conditions. As examples, Figures 11, 12, 13 and 14(a) show the time signals measured in each state of the motor bearing and processed by high-pass filter (5KHz cut-off frequency). Figures 11, 12, 13 and 14(b) show the part of time signals. Figures 11, 12, 13 and 14(c) show the contour graphs of the spectra processed by the PWVD in each state. Figures 11, 12, 13 and 14(d) show the RCI of the spectra expressed by *I_q_* under each state. From the Figures 11, 12, 13 and 14, the values of the RCI in the feature spectra caused by the defect are larger than those in the spectra without a defect, and the shapes of the feature spectra are different in each state. We can extract the instantaneous feature spectra of the outer-race defect, inner-race defect, and roller element defect states in the positions A-A, B-B, and C-C, respectively, where the RCI values are maximum. The extracted feature spectra are instantaneous and not correlated with the rotation speed and load, thus, they can be used to detect the machine faults and identify the fault types under variable rotation speed and load.

After extracting the feature spectra from the time-frequency domain by the RCI, the SPs that can express the characteristics of these feature spectra are calculated by Equations (6)–(11). Two SPs that have the highest sensitivity at each sequential diagnostic step are selected by the DI. As an example, parts of the DI values of the SPs and selection results are shown in Table 2.

In the first diagnostic step, P1 and P4 can distinguish the normal and outer-race defect states more easily than the other SPs because the DI values of P_1_ and P_4_ for distinguishing these states are larger than those of the other SPs. The SPs for the other diagnostic steps can be selected in a similar manner. The SPs selected by the DI are input into the ACO clustering model. After approximately 200 iterations, the ACO converged to the optimum clustering centers. Table 3 provides the clustering centers for each sequential diagnostic step.

Using the clustering centers shown in Table 3 and the method introduced in Section 4, the SSPs for each sequential diagnostic step can be obtained. The calculation Formulas and values of the SSPs for each sequential diagnostic step are shown in Tables 4 and 5, respectively.

### 6.3. Sequential Condition Diagnosis by Possibility Theory

In this section, we show the method based on possibility theory and sequential inference to identify the conditions of the motor bearing under variable operating conditions. According to the sequential inference, the whole diagnostic process is divided into three steps. First, the normal state is distinguished from the abnormal states using the corresponding possibility of the SSPs. Second, the outer-race defect state is distinguished from the other abnormal states using the corresponding possibility of the SSPs. Finally, the inner-race defect and roller element defect states are distinguished using the corresponding possibility of the SSPs. The diagnostic process and results are presented as follows.

Step 1 of the sequential diagnosis and verification:

The possibility functions *μ_n_*(x), *μ_o_*(x), *μ_i_*(x) and *μ_r_*(x) of the SSPs in the normal, the outer-race defect, inner-race defect and roller element defect levels, are calculated by Equations (27) and (28), respectively. The possibility function of the SSPs in other abnormal level is expressed with *μ_a_*(x). The membership function between the normal and the abnormal states is calculated by:

(31) $$\mu_{n1}(\text{x})+\mu_{o}(\text{x})+\mu_{a}(\text{x})=1$$

(32) $$\mu_{n2}(\text{x})+\mu_{i}(\text{x})+\mu_{a}(\text{x})=1$$

(33) $$\mu_{n3}(\text{x})+\mu_{r}(\text{x})+\mu_{a}(\text{x})=1.$$

**Figure 15.**
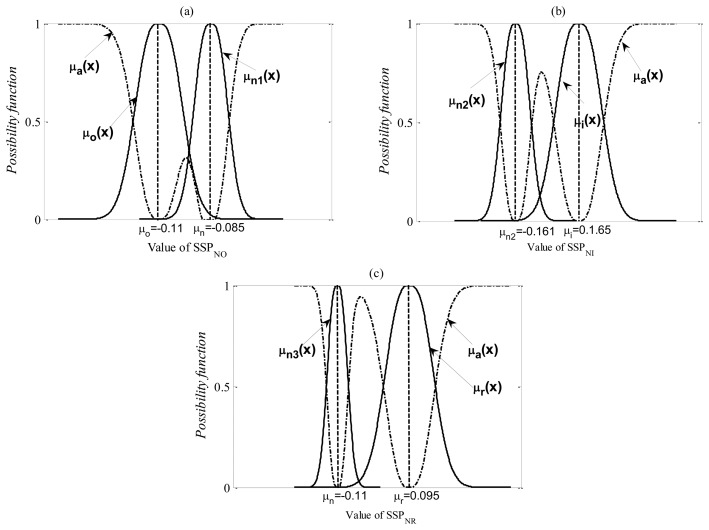
Membership functions for the first diagnostic step.

The membership functions of each level for the first diagnostic stage are shown in Figure 15.

**Figure 16.**
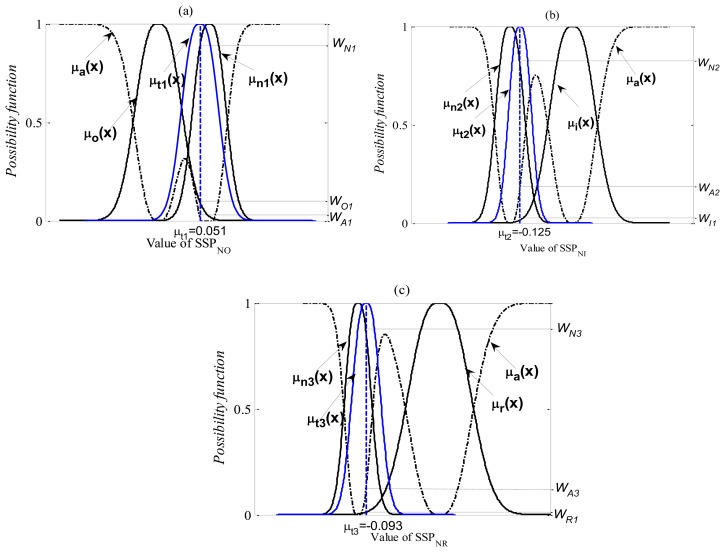
The practical diagnosis examples for the test 1.

**Figure 17.**
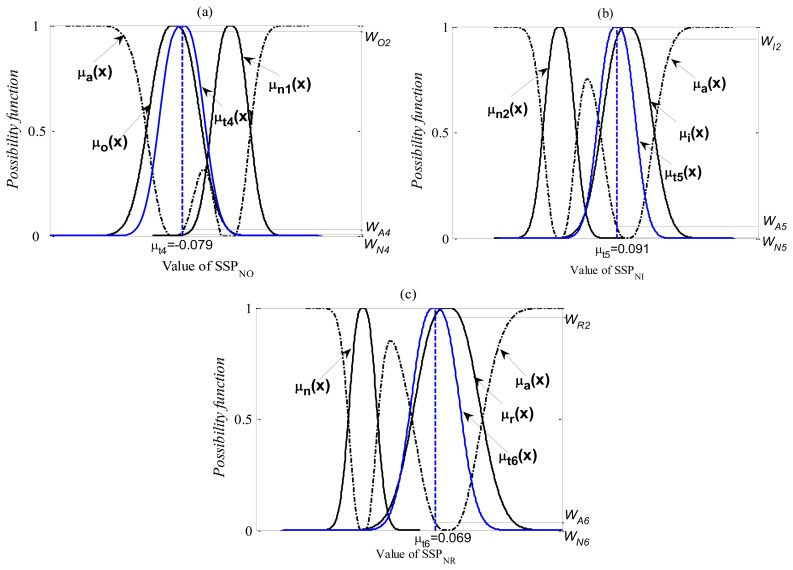
The practical diagnosis examples for the test 2.

In order to verify the diagnostic capability of the proposed method, all of the verification signals, which are measured in each known state had not been used for the pre-calculated possibility function are used. Figures 16 and 17 show the practical diagnosis examples for the first diagnostic step. Here, *μ_t_*_1_(*x*), *μ_t_*_2_(x) and *μ_t_*_3_(x) are the possibility distributions of the SSPs calculated from the verifying signals in the normal state. *μ_t_*_4_(x), *μ_t_*_5_(x) and *μ_t_*_6_(x) are the possibility distributions of the SSPs calculated from the verifying signals in the outer-race defect, inner-race defect and roller element defect states, respectively. The matching degree for each state can be obtained by Equation (30). These degrees are normalized as follows:

(34) $${\text{W}}_{\text{Ni}}+{\text{W}}_{\text{Ai}}+{\text{W}}_{\text{Oi}}=1$$

(35) $${\text{W}}_{\text{Ni}}+{\text{W}}_{\text{Ai}}+{\text{W}}_{\text{Ii}}=1$$

(36) $${\text{W}}_{\text{Ni}}+{\text{W}}_{\text{Ai}}+{\text{W}}_{\text{Ri}}=1$$

The verification results for the first diagnostic step are as follows:
Test 1
N: O
Possibility of the normal state: *W_N1_* = 88.7%.Possibility of the outer-race defect state: *W_O1_* = 9.4%.Possibility of other abnormal state: *W_A1_* = 1.9%.Judgment: normal state.N:I
Possibility of the normal state: *W_N2_* = 83.8%.Possibility of the inner-race defect state: *W_I1_* =2.4%.Possibility of other abnormal state: *W_A2_* = 13.8%.Judgment: normal state.N:R
Possibility of the normal state: *W_N3_* = 86.8%Possibility of the roller element defect state: *W_R1_* = 1.7%Possibility of other abnormal state: *W_A3_* = 11.5%.Judgment: normal state.Test 2
N: O
Possibility of the normal state: *W_N4_* = 0.1%.Possibility of the outer-race defect state: *W_O2_* = 96.9%.Possibility of other abnormal state: *W_A4_* = 3%.Judgment: outer-race defect state.N: I
Possibility of the normal state: *W_N5_* = 0.5%.Possibility of the inner-race defect state: *W_I2_* = 90.5%.Possibility of other abnormal state: *W_A5_* = 9%.Judgment: inner-race defect l state.N: R
Possibility of the normal state: *W_N6_* = 0%Possibility of the roller element defect state: *W_R2_* = 97.5%Possibility of other abnormal state: *W_A6_* = 2.5%.

Judgment: roller element defect state.

The other fault states can also be distinguished sequentially in a similar manner. The verification results for Steps 2 and 3 are as follows:

Step 2 of the sequential diagnosis and verification:
Test 3
O: I
Possibility of the outer-race defect state: *W_O3_* = 86.4%.Possibility of the inner-race defect state: *W_I3_* = 13.5%.Possibility of other abnormal state: *W_A7_* = 0.1%.Judgment: outer-race defect state.O: R
Possibility of the outer-race defect state: *W_O4_* = 76.2%.Possibility of the roller element state: *W_R3_* = 3.8%.Possibility of other abnormal state: *W_A7_* = 20%.

Judgment: outer-race defect state.


Test 4
O: I
Possibility of the outer-race defect state: *W_O5_* = 0%.Possibility of the inner-race defect state: *W_I4_* = 97.5%.Possibility of other abnormal state: *W_A8_* = 2.5%.Judgment: inner -race defect state.O: R
Possibility of the outer-race defect state: *W_O6_* = 0%.Possibility of the roller element defect state: *W_R4_* = 97.8%.Possibility of other abnormal state (A): *W_A9_* = 2.2%.Judgment: roller element defect state.
Step 3 of the sequential diagnosis and verification:
Test 5 I: R
Possibility of the inner-race defect state: *W_I5_* = 77.5%.Possibility of the roller element defect state: *W_R5_* = 22.5%.Possibility of other abnormal state: *W_A11_* = 0%.Judgment: inner-race defect state.Test 6 I: R
Possibility of the inner-race defect state: *W_I6_* = 33.3%.Possibility of the roller element state: *W_R6_* = 66.7%.Possibility of other abnormal state: *W_A12_* = 0%.Judgment: roller element defect state.

According to these diagnosis results, the normal, outer-race defect, inner-race defect and roller element defect states of the motor bearing can be automatically and correctly identified using the possibilities of the SSPs and other diagnosis methods proposed in this paper.

## 7. Conclusions

In this paper, we propose a new fault diagnosis method for motor roller bearings which operates under variable conditions, namely, their rotation speed and operating load are always changing. The feature of each machine state could be expressed by the PWVD. The extraction method for feature spectra was proposed using the RCI, by which the instantaneous feature spectra from time-frequency analysis was automatically extracted by a computer in order to identify the conditions of a machine under the variable operating conditions. The instantaneous feature spectra caused by the local defects were clearly expressed, and they could reflect the characteristic of the signal or the motor bearing condition. Moreover, the excellent SSPs for expressing the characteristics of the feature spectra were obtained by ACO clustering algorithm. The diagnostic sensitivity of the SSPs was greater than the original SPs, and the conditions of the machine could be identified more easily. A fuzzy diagnosis method based on sequential inference and possibility theory was also proposed, by which the conditions of the machine can be identified sequentially. It is proved that the methods proposed in this paper were effective by applying them to the motor roller bearing diagnosis.

## Figures and Tables

**Table 1.** Bearing information for verification.

**Contents**	**N205**	**NU205**
Bearing outer diameter	52 mm	52 mm
Bearing inner diameter	25 mm	25 mm
Bearing width	15 mm	15 mm
Bearing roller diameter	7 mm	7 mm
The number of the rollers	10	11
Contact angle	0 rad	0 rad
Outer-race defect(width × depth)	0.3 × 0.25 mm Early stage	
Rolling element defect (width × depth)	0.5 × 0.15 mm Early stage	
Inner-race defect (width × depth)		0.3 × 0.25 mm Early stage

**Table 2.** DI values of the SPs for each sequential diagnosis step.

**DI Values of Each SP**						
	P_1_	P_2_	P_3_	P_4_	P_5_	P_6_
For the diagnosis first step:						
N:O	1.96	1.79	1.02	2.01	0.42	0.85
N:I	2.12	2.25	0.24	1.21	0.47	0.67
N:R	2.32	2.28	0.47	1.64	0.46	0.58
For the diagnosis second step:						
O:I	1.95	1.53	0.53	2.07	0.22	0.23
O:R	1.86	1.74	1.03	1.78	0.40	0.17
For the diagnosis third step:						
I:R	1.39	1.57	0.81	1.21	0.93	0.92

**Table 3.** Clustering centers for each sequential diagnosis step.

**Clustering Centers**			**Space of SPs**
For the first diagnosis step:			
N:O	N(0.46,0.91)	O(0.32,0.77)	P1, P4
N:I	N(0.46,1.84)	I(0.56,2.15)	P1, P2
N:R	N(0.46,1.84)	R(0.62,1.97)	P1, P2
For the second diagnosis step:			
O:I	O(0.32,0.77)	I(0.56,0.88)	P1, P4
O:R	O(0.32,0.77)	R(0.62,0.87)	P1, P4
For the third diagnosis step:			
I:R	I(0.56,2.15)	R(0.62,1.97)	P1, P2

**Table 4.** SSP Formula for each sequential diagnosis step.

**Synthetic Symptom Parameters (SSPs)**	
For the first diagnosis step:	
N:O	$SSP_{NO}=m\sqrt{{\left(P_{1}+0.93P_{4}-1.17\right)}^{2}+{\left(0.93P_{1}+0.82P_{4}-1.1\right)}^{2}}/1.87$
N:I	$SSP_{NI}=m\sqrt{{\left(P_{1}+3.1P_{2}-6.7\right)}^{2}+{\left(3.1P_{1}+9.6P_{2}-20.7\right)}^{2}}/10.6$
N:R	$SSP_{NR}=m\sqrt{{\left(P_{1}+0.81P_{2}-2.1\right)}^{2}+{\left(0.81P_{1}+0.66P_{2}-1.7\right)}^{2}}/1.66$
For the second diagnosis step:	
O:I	$SSP_{OI}=m\sqrt{{\left(P_{1}+0.48P_{4}-0.84\right)}^{2}+{\left(0.48P_{1}+0.23P_{4}-0.4\right)}^{2}}/1.23$
O:R	$SSP_{OR}=m\sqrt{{\left(P_{1}+0.34P_{4}-0.76\right)}^{2}+{\left(0.34P_{1}+0.1P_{4}-0.26\right)}^{2}}/1.12$
For the third diagnosis step:	
I:R	$SSP_{IR}=m\sqrt{{\left(P_{1}+3P_{2}-5.6\right)}^{2}+{\left(9P_{2}-3P_{1}-16.8\right)}^{2}}/10$

**Table 5.** Values of SSP for each sequential diagnosis step.

SSPs																									**μ**	**σ**
N:O	N	0.102	0.076	0.078	0.022	0.071	0.056	0.124	0.122	0.065	0.125	0.130	0.115	0.096	0.121	0.021	0.056	0.068	0.052	0.091	0.131	0.053	0.022	0.110	0.083	0.035
	O	−0.137	−0.122	−0.127	0.054	0.023	−0.079	−0.103	−0.138	−0.160	−0.091	−0.093	−0.123	−0.160	−0.100	−0.147	−0.069	−0.145	−0.123	−0.126	−0.137	−0.146	−0.133	−0.160	−0.111	0.052
N:I	N	−0.112	−0.215	−0.123	−0.191	−0.138	−0.215	−0.202	−0.204	−0.193	−0.147	−0.130	−0.133	−0.141	−0.122	−0.112	−0.192	−0.157	−0.215	−0.115	−0.208	−0.203	−0.112	−0.138	−0.162	0.039
	I	0.178	0.208	0.225	0.023	0.112	0.143	0.062	0.094	0.225	0.208	0.112	0.085	0.054	0.239	0.208	0.312	0.439	0.143	0.292	0.023	0.062	0.167	0.178	0.165	0.099
N:R	N	−0.066	−0.105	−0.117	−0.126	−0.107	−0.142	−0.123	−0.140	−0.105	−0.071	−0.055	−0.119	−0.090	−0.106	−0.116	−0.155	−0.122	−0.116	−0.104	−0.117	−0.131	−0.120	−0.076	−0.110	0.024
	R	0.128	0.083	−0.065	0.168	0.138	−0.092	0.084	0.088	0.087	0.017	0.118	−0.007	0.150	0.154	0.088	0.118	0.138	0.157	0.154	0.098	0.087	0.150	0.138	0.095	0.068
O:I	O	−0.162	−0.176	−0.191	−0.097	−0.129	−0.088	−0.118	−0.162	−0.088	−0.102	−0.106	−0.172	−0.112	−0.115	−0.099	−0.119	−0.088	−0.201	−0.088	−0.160	−0.172	−0.156	−0.191	−0.134	0.038
	I	0.016	0.014	0.084	0.075	0.009	0.074	0.070	0.089	0.271	0.060	0.084	0.074	0.089	0.271	0.016	0.213	0.271	0.294	0.183	0.237	0.236	0.210	0.074	0.131	0.095
O:R	O	−0.201	−0.125	−0.142	−0.133	−0.138	−0.124	−0.212	−0.202	−0.201	−0.138	−0.142	−0.231	−0.149	−0.152	−0.135	−0.156	−0.231	−0.210	−0.124	−0.199	−0.211	−0.195	−0.231	−0.173	0.038
	R	0.092	0.037	0.069	0.035	0.087	0.057	0.087	0.237	0.035	0.086	0.033	0.094	0.238	0.200	0.191	0.215	0.254	0.036	0.210	0.254	0.253	0.218	0.238	0.142	0.086
I:R	I	0.058	0.086	−0.141	0.014	−0.051	−0.062	−0.029	−0.048	−0.112	−0.130	−0.070	−0.050	−0.007	−0.124	−0.113	−0.178	−0.263	−0.112	−0.179	−0.113	−0.179	−0.196	−0.102	−0.091	0.082
	R	0.164	0.126	0.229	0.270	0.158	0.246	0.156	0.099	0.123	0.127	0.089	0.122	0.086	0.106	−0.004	−0.011	0.080	0.069	0.020	−0.065	0.042	−0.005	0.042	0.099	0.083
